# Multidimensional Approach Assessing the Role of Interleukin 1 Beta in Mesial Temporal Lobe Epilepsy

**DOI:** 10.3389/fneur.2021.690847

**Published:** 2021-08-05

**Authors:** Renato O. Santos, Rodrigo Secolin, Patrícia G. Barbalho, Mariana S. Silva-Alves, Marina K. M. Alvim, Clarissa L. Yasuda, Fábio Rogerio, Tonicarlo R. Velasco, Americo C. Sakamoto, Antonio L. Teixeira, Fernando Cendes, Claudia V. Maurer-Morelli, Iscia Lopes-Cendes

**Affiliations:** ^1^Department of Translational Medicine, University of Campinas, Campinas, Brazil; ^2^Brazilian Institute of Neuroscience and Neurotechnology, Campinas, Brazil; ^3^Department of Neurology, University of Campinas, Campinas, Brazil; ^4^Department of Pathology, University of Campinas, Campinas, Brazil; ^5^Department of Neuroscience and Behavioral Science, Ribeirão Preto Medical School, University of São Paulo, Ribeirão Preto, Brazil; ^6^Department of Internal Medicine, Federal University of Minas Gerais, Belo Horizonte, Brazil

**Keywords:** mesial temporal sclerosis, hippocampal atrophy, association study, gene expression, neuroinflammation

## Abstract

We aimed to investigate the role of interleukin-1 beta (IL-1β) in the mechanisms underlying mesial temporal lobe epilepsy with hippocampal sclerosis (MTLE+HS). We assessed a cohort of 194 patients with MTLE+HS and 199 healthy controls. Patients were divided into those with positive and negative antecedent febrile seizures (FS). We used a multidimensional approach, including (i) genetic association with single nucleotide polymorphisms (SNPs) in the *IL1B* gene; (ii) quantification of the *IL1B* transcript in the hippocampal tissue of patients with refractory seizures; and (iii) quantification of the IL-1β protein in the plasma. We found a genetic association signal for two SNPs, rs2708928 and rs3730364^*^C in the *IL1B* gene, regardless of the presence of FS (adjusted *p* = 9.62e–11 and 5.14e–07, respectively). We found no difference between *IL1B* transcript levels when comparing sclerotic hippocampal tissue from patients with MTLE+HS, without FS, and hippocampi from autopsy controls (*p* > 0.05). Nevertheless, we found increased IL-1β in the plasma of patients with MTLE+HS with FS compared with controls (*p* = 0.0195). Our results support the hypothesis of a genetic association between MTLE+HS and the *IL1B* gene

## Introduction

Mesial temporal lobe epilepsy with hippocampal sclerosis (MTLE+HS) is the most common and usually pharmacoresistant form of focal epilepsy in the adult population (1). In addition, MTLE+HS has been associated with the presence of an initial precipitating injury (IPI) in some patients, including trauma, *status epilepticus*, infections, and childhood febrile seizures (FS) (2).

Clinical and experimental evidence indicates a link between initial precipitating injuries (IPIs) and neuroinflammation, leading to the development of epilepsy (3–7). Furthermore, it has been suggested that the interleukin-1 family may play a role in the development of FS and subsequently in MTLE+HS (8, 9). Among the interleukin-1 family, interleukin-1 beta (IL-1β) is the primary cytokine responsible for intermediating human febrile responses (7, 8). Indeed, many studies have shown a link between IL-1β and MTLE (10–16). The use of an anticytokine compound to block IL-1β synthesis in the kindling animal model suggests that IL-1β is involved in kindling progression (17). In addition, a subtle increase in the levels of IL-1β was observed in plasma from children with febrile *status epilepticus*, as part of the FEBSTAT study (14).

Altogether, these findings raise the hypothesis that genetic variation in the *IL1B* gene could lead to abnormal levels of *IL1B* RNA transcripts and consequently of IL-1β protein levels, which, in turn, could influence the development of MTLE+HS (18). In fact, a previous study has identified a genetic association between the single nucleotide polymorphism (SNP) rs16944 and patients with MTLE+HS in the Japanese population. This SNP is located in the promoter region of the *IL1B* (c.-511C>T), suggesting that this change may be associated with abnormal levels of IL-1β (10, 19). However, these results were not replicated in subsequent studies in patients of European and Chinese origins (19–23). More recently, a systematic meta-analysis including 17 studies of European and Chinese children with FS suggested that the SNP rs16944 on *IL1B* could be a risk factor for FS and the subsequent development of MTLE+HS (24).

In this context, we aimed to assess a cohort of patients with MTLE+HS to explore the role of IL-1β at three different molecular domains: (i) a genetic association study with polymorphisms covering the *IL1B* gene; (ii) quantification of the *IL1B* transcript in the hippocampal tissue from patients with pharmacoresistant MTLE+HS; and (iii) IL-1β protein quantification in the plasma of patients with MTLE+HS.

## Materials and Methods

We included 194 unrelated patients with MTLE+HS from two clinical centers. One hundred and seventy patients were followed at the epilepsy clinic of the University of Campinas (UNICAMP) University Hospital and 24 at the epilepsy clinic of the University of São Paulo at Ribeirão Preto (USP-RP) University Hospital. This study was approved by the research ethics committee of the UNICAMP and USP-RP, and written informed consent was obtained from all participants.

### Characterization of the Patients

Only adults were included in this study; the mean age of the patients was 45.7 years old, ranging from 23 to 90 years of age. MTLE+HS was confirmed in all the patients according to the International League Against Epilepsy (ILAE) criteria (25). Clinical evaluation was performed by experienced neurologists in the management of the patients with epilepsy. All the patients were interviewed using a structured questionnaire to collect information about age, the onset of epilepsy, detailed seizure semiology, history of FS, other IPIs, family history of epilepsy, and the number of antiseizure medications used. A positive antecedent of FS was registered if confirmed by a relative or in the presence of medical records. In addition, all the patients had a neurological examination, serial interictal electroencephalograms, and high-resolution magnetic resonance imaging (MRI) with a specific epilepsy protocol to identify hippocampal atrophy or other MRI signs of HS (26).

Patients with dual pathology (HS associated with other structural lesions on MRI), mental retardation, associated progressive neurologic diseases, autoimmune diseases, cancer, or major chronic debilitating conditions such as renal failure or hepatic failure were not included in this study.

### Characterization of the Control Group

The control group included 199 unrelated healthy individuals, mainly unrelated spouses of the patients. None of the control subjects had an antecedent of epilepsy or FS in their own family (blood relatives). In addition, 41 control individuals had an MRI to confirm the absence of hippocampal abnormalities.

### MRI Acquisition

The MRIs were acquired using a 3T scanner (Philips Achieva) in coronal, sagittal, and axial planes, as well as 3D T1 high-resolution with 1-mm^3^ voxel size. The coronal images for visual analysis were obtained perpendicular to the long axis of the hippocampus and included 1-mm T1-weighted, 3-mm T1-inversion recovery, 3-mm fast spin-echo T2-weighted, 3-mm T2-weighted multiecho, and 1- or 3-mm FLAIR (fluid-attenuated inversion recovery). MRI signs of HS include hippocampal atrophy, increased T2 and FLAIR signal intensity, and abnormal shape and internal structure of the hippocampus (27), which were defined by visual analysis and confirmed by hippocampal volumetry and T2 relaxometry as described previously (26).

### SNP Genotyping

Genomic DNA was isolated from peripheral blood from 194 patients with MTLE+HS and 199 controls using the phenol-chloroform method (28). DNA samples were quantified using a spectrophotometer (NanoVue, GE Healthcare, Pittsburgh, PA, USA), and samples with a 260/280 ratio ≥ 1.7 were used in the study.

We selected SNPs located within the *IL1B* gene by the SNPBROWSER^TM^ 4.0 software (Applied Biosystems, Foster City, CA, USA) based on the SNPs from the HapMap project (29), a minimum allele frequency (MAF) > 0.01, and linkage disequilibrium (*r*^2^) > 0.8. To verify the presence of population stratification, we genotyped additional 23 SNPs as a genomic control on chromosome 2 (Supplementary Table 1). These were randomly selected using an in-house algorithm developed in the R software (30). These SNPs were located outside the *IL1B* region, with a minimum distance of 0.3 cM (300 kb) between each other, to avoid possible linkage disequilibrium (31). We focused on using SNPs on chromosome 2 for the genomic control because previous studies showed differences in fixation index (Fst) values among chromosomes, and the presence of natural selection imprint observed in many regions of the genome could create significant variation in Fst, a phenomenon that could lead to biased global Fst values (32).

SNPs were genotyped using the SNPlex^TM^ Genotyping System 48-plex technology (Applied Biosystems). Allelic discrimination was performed through a capillary electrophoresis analysis with the Applied Biosystems 3130xl DNA Analyzer and the GeneMapper^TM^ 4.0 software. Also, we genotyped the rs16944 allele by polymerase chain reaction (PCR) amplification of a 204-bp fragment and restriction fragment length polymorphism (RFLP) using the following primers: 5′-GGCTAGGGTAACAGCACCTG-3′ and 5′-TGAGGGTGTGGGTCTCTACC-3′. PCR conditions were as follows: a denaturing step of 94°C for 10 min, then 35 cycles of 94°C for 20 s, 55°C for 20 s, 72°C for 40 s, and a final incubation at 72°C for 10 min. RFLP analysis was performed using 5 U *Dde*I (Thermo Scientific, San Jose, CA, USA) at 37°C overnight, and PCR products were run on a 12% polyacrylamide gel followed by ethidium bromide staining. PCR products were cut in three fragments of 131, 42, and 31 bp for the rs16944^*^A allele or two fragments of 131 and 73 bp for the rs16944^*^G allele.

### *IL1B* RNA Quantification in Hippocampal Tissue

To investigate the *IL1B* RNA levels in sclerotic tissue, we used hippocampal specimens from four patients with MTLE+HS, who underwent a standard anteromedial temporal resection to treat pharmacoresistant seizures at the UNICAMP University Hospital. None of the four patients with MTLE+HS presented antecedent FS. We compared these samples with six autopsy controls, whose cause of death was other than central nervous system diseases. All tissues were evaluated by a neuropathologist, who confirmed hippocampal sclerosis in samples from patients, and no histopathological abnormalities in autopsy tissue used as a control.

The hippocampal specimens were frozen in liquid nitrogen immediately after resection and stored at −80°C until processing. Total RNA was obtained using a TRIzol® Reagent (Invitrogen, Carlsbad, CA, USA). RNA samples were quantified by a spectrophotometer (NanoVue GE Health Care, Chicago, IL, USA), and samples that met the requirement of a 260/280 ratio ≥ 1.9 were used in the study. The RNA integrity was checked on a 1% agarose gel. According to the instructions of the manufacturer, 1 μg of total RNA was reverse-transcribed into complementary DNA (cDNA) using SuperScript III^TM^ Reverse Transcriptase (Invitrogen, Carlsbad, CA, USA).

*IL1B* transcript quantification was carried out using a TaqMan® Gene Expression Assay (Hs01555410_m1) in an ABI 7500 Real-Time PCR system (Applied Biosystems, Waltham, MA, USA) using *SYP* (synaptophysin, Hs00300531_m1) and *SDHA* (succinate dehydrogenase, subunit A, Hs00417200_m1) as endogenous control genes because they were previously validated for the normalization of target genes in human hippocampal samples obtained from patients with MTLE (33). Reactions were performed in triplicate, and the relative quantification (RQ) was calculated according to the equation RQ = 2^−Δ*ΔCT*^ (34). Data were analyzed using the SDS 7500 software (Applied Biosystems, Waltham, MA, USA).

### IL-1β Protein Quantification in Plasma

We obtained plasma samples from 108 patients with MTLE+HS and 75 controls. Of these, 90 patients had an antecedent of FS. Samples were collected and analyzed blindly regarding clinical status. Also, all samples were collected in the morning to avoid influences from the circadian cycle. We did not collect the blood in patients presenting any symptoms of infectious disease. Blood samples were centrifuged for 10 min at 3,000 rpm at 4°C, and plasma was collected and stored in a −80°C freezer until analyzed. IL-1β levels in plasma were measured by enzyme-linked immunosorbent assay (ELISA) using a high sensitivity immunoassay kit (Human IL-1Beta Quantikine High Sensitivity ELISA, R&D Systems, Minneapolis, MN, USA). Samples were analyzed in duplicate, and concentrations are expressed as pg/ml. The lower detection limit for IL-1β is 0.023 pg/ml.

We compared the proportion of patients who had seizures <24 h and more than 24 h before the blood collection and the proportion of patients with depressive symptoms in the patients' subgroups with and without FS using Fisher's exact test; these variables could affect the IL-1β plasma levels. In addition, we assessed depressive symptoms by the Beck Depression Inventory (BDI-II) scores.

### Statistical Analysis

We compared patients and controls for sex distribution using Fisher's exact test and mean age using Student's *t*-test, both performed in the R software (www.r-project.org). The SNPs were filtered by minimum allele frequency (MAF > 0.01) and Hardy–Weinberg disequilibrium (*p* < 0.01) by the PLINK v1.9 software (35). We also evaluated the linkage disequilibrium in terms of *r*^2^ among SNPs by the HAPLOVIEW software (36).

Population stratification is a known phenomenon that could lead to biased genetic association results (37, 38). Therefore, to assess whether our sample of patients and controls presents population stratification, we performed an analysis of molecular variance (AMOVA) using the ARLEQUIN software (39), based on the 23 genomic control SNPs. The AMOVA approach divides the source of genetic variance (σ^2^) into two components: within-groups and between-groups. Under a null hypothesis, the samples were obtained from a global population, and the variation is due to random sampling in the construction of populations. Thus, we would expect a high variation within-groups (σ^2^ = 100%) and no variation between-groups (σ^2^ = 0%). On the other hand, under the alternative hypothesis of population stratification, each group was obtained from different populations, and we would expect a low variation within-groups (σ^2^ <100%) and high variation between-groups (σ^2^ > 0%) (40).

The genetic association between SNPs in *IL1B* and MTLE+HS was estimated using an additive logistic regression model by the PLINK v1.9 software (35). Because we performed two comparisons (controls vs. MTLE+HS with FS and controls vs. MTLE+HS without FS), we adjusted *p*-values with the Bonferroni correction to account for multiple comparisons. We also estimated the effect size in terms of an odds ratio (OR) with a 95% confidence interval (CI). To evaluate the sensitivity of our sample to detect an association, we estimated the effect size in terms of OR using the G^*^POWER software (41), including the following parameters: logistic regression model; two-sided analysis; a level of significance α = 0.00625 (adjusted by Bonferroni correction); statistical power 1–β = 0.80; and total sample = 393.

We used the Wilcoxon rank-sum test, using the *Wilcox.test* function in the R software, to compare the RQ of the *IL1B* RNA transcript from the hippocampal tissue between patients and controls for both endogenous controls (*SYP* and *SDHA*). We also used the Wilcoxon rank-sum test to compare the IL-1β plasma levels in two scenarios: MTLE+HS with FS and controls and between MTLE+HS without FS and controls. Because we performed two comparisons, *p*-values were adjusted by the Bonferroni correction. The effect size was also calculated in *R*, including the 95% CI, using the *Wilcox.test* function.

## Results

### Subjects

Demographic and clinical data are shown in Table 1. Among the patients, the mean age was 45.7 years old, ranging from 23 to 90 years old (standard deviation [SD] = 11.2), and the mean age at seizure onset was 11.14 years old, ranging from 1 to 53 years old (standard deviation [SD] = 9.53). Patients with MTLE+HS were found to be older than controls (Student's *t*-test *p* = 0.011). Although we did not observe differences in the sex distribution between the two groups (Fisher's exact test *p* = 0.361), we observe an imbalance in the female/male ratio between patients with MTLE+HS (ratio = 1.18) and control individuals (ratio = 0.7). Therefore, we included age and sex as covariates to adjust the logistic regression model. We also identified 40 patients with an antecedent of FS (20.6%). All 194 patients presented hippocampal sclerosis, and all 41 normal controls who had MRIs did not present any signs of hippocampal sclerosis.

**Table 1:** Descriptive statistics of patients with MTLE+HS and controls ascertained for the genetic association study.

**Variable**		**MTLE+HS with FS (*n* = 40)**	**MTLE+HS without FS (*n* = 126)**	**MTLE+HS FS unknown (*n* = 28)**	**Controls (*n* = 199)**	***p*-value**
Sex	Female	23 (57.5%)	73 (57.9%)	9 (32.1%)	82 (20.9%)	0.361
	Male	17 (42.5%)	53 (42.1%)	19 (67.9%)	117 (29.8%)	
Mean age (years)		45.7 (SD = 11.2)	52.4 (SD = 10.9)	–^*^	47.15 (SD = 16.61)	0.011
Mean age at onset (years)		8.4 (SD = 10.3)	12.2 (SD = 9.2)	–^*^	–	–

*FS, febrile seizures; MTLE+HS, mesial temporal lobe epilepsy with hippocampal sclerosis; SD, standard deviation*.

* *We did not estimate mean age and mean age at onset of epilepsy in the group of patients with an unknown antecedent of FS because only three patients had age and age at onset available*.

*The p-value refers to the comparison between all patients with MTLE+HS and controls*.

### *IL1B* Candidate Gene Association Analysis

We found that our cohort can detect a genetic association with 80% statistical power with an OR <0.63 (protective allele effect) or an OR > 1.58 (increased risk allele effect). Furthermore, the AMOVA results also showed that 99.35% of the variation component was observed within groups, and 0.65% was observed between groups (Table 2), indicating that the sample does not present population stratification. Hence, the sample provides unbiased genetic association results.

**Table 2:** Summary of the AMOVA results for the population structure analysis.

**Variation source**	**SSQ**	**VC**	**VC (%)**	**Fst**
Between-groups	11.340	0.0263	0.65	0.00647
Within-groups	2,455.741	4.0212	99.35	
Total	2,467.081	4.0475	100.00	

*AMOVA, analysis of molecular variance; Fst, fixation index; SSQ, sum of squares; VC, variance components*.

The eight SNPs genotyped in the *IL1B* gene (Table 3) presented a mean genotype call rate of 90.87%. Two SNPs presented Hardy–Weinberg disequilibrium (rs3917365, *p* = 8.34e-07; rs1143633, *p* = 2.07e-24) and were excluded from the logistic regression analysis. In addition, SNPs rs3917368 and rs1143646 presented linkage disequilibrium (*r*^2^ = 0.96). As shown in Table 3, the SNP rs16944—located in the promoter region of the *IL1B* gene—showed no association with MTLE+HS and a small effect for both MTLE+HS with FS (rs16944^*^T: OR = 1.06, 95% CI = 0.76–1.46, *p* = 1.000) and MTLE+HS without FS (rs16944^*^T: OR = 0.98, 95% CI = 0.70–1.39, *p* = 1.000). By contrast, two other SNPs showed evidence of association with the MTLE+HS with the FS phenotype (rs2708928, adjusted *p* = 9.62e^−11^; rs3730364, adjusted *p* = 5.14e^−07^). These two SNPs presented a protective effect (rs2708928^*^G: OR = 0.10, 95% CI = 0.05–0.20; rs3730364^*^T: OR = 0.38, 95% CI = 0.26–0.54), while other alleles were associated with an increased risk effect (rs2708928^*^C: OR = 9.66, 95% CI = 5.01–18.60; rs3730364^*^C: OR = 2.66, 95% CI = 1.86–3.79). Additional analyses, in which patients with MTLE+HS with FS were removed, showed similar results for the SNPs rs2708928 (adjusted *p* = 2.05e^−10^) and rs3730364 (adjusted *p* = 1.34e^−05^), including similar estimates for the OR (Table 3).

**Table 3:** Results of the logistic regression for the eight SNPs analyzed within the *IL1B* gene.

**SNP**	**BP**	**HWD p**	**Allele**	**Allele frequency**		**Including MTLE+HS with FS**			**Removing MTLE+HS with FS**		
				**MTLE+HS**	**Controls**	**OR**	**95% CI**	**Adjusted *p***	**OR**	**95% CI**	**Adjusted *p***
rs3917368	112,825,205	1.000	T	0.306	0.356	0.83	0.60–1.16	1.000	0.83	0.57–1.19	1.000
			C	0.694	0.644	1.20	0.86–1.67		1.21	0.84–1.75	
rs3917365	112,828,892	8.34e-07	G	0.668	0.865	–	–	–	–	–	–
			A	0.332	0.135	–	–		–	–	
rs1143643	112,830,725	0.282	T	0.292	0.347	0.80	0.58–1.11	1.000	0.83	0.58–1.18	1.000
			C	0.708	0.653	1.25	0.90–1.74		1.21	0.85–1.72	
rs1143633	112,832,890	2.07e-24	T	0.565	0.697	–	–	–	–	–	–
			C	0.435	0.303	–	–		–	–	
**rs2708928**	**112,834,661**	**0.021**	**C**	**0.203**	**0.037**	**9.66**	**5.01–18.60**	**9.62e** ^**−11**^	**10.64**	**5.31–21.33**	**2.05e** ^**−10**^
			**G**	**0.797**	**0.963**	**0.10**	**0.05–0.20**		**0.09**	**0.05–0.19**	
**rs3730364**	**112,836,014**	**0.143**	**C**	**0.673**	**0.414**	**2.66**	**1.86–3.79**	**5.14e** ^**−07**^	**2.51**	**1.72–3.66**	**1.34e** ^**−05**^
			**T**	**0.327**	**0.586**	**0.38**	**0.26–0.54**		**0.40**	**0.27–0.58**	
rs16944 (−511C>T)	112,837,290	0.445	T	0.391	0.373	1.06	0.76–1.46	1.000	0.98	0.70–1.39	1.000
			C	0.609	0.627	0.95	0.68–1.31		1.02	0.72–1.44	
rs13032029	112,842,838	1.000	T	0.459	0.443	1.04	0.76–1.43	1.000	0.94	0.67–1.32	1.000
			C	0.541	0.557	0.96	0.70–1.32		1.07	0.76–1.50	

*The results are presented with and without 40 patients with FS. The p-values were adjusted by the Bonferroni correction. CI, confidence interval; FS, febrile seizures; HWD, Hardy–Weinberg disequilibrium; IL1B, interleukin 1 beta; MTLE+HS, mesial temporal lobe epilepsy with hippocampal sclerosis; OR, odds ratio; SNP, single nucleotide polymorphism. We highlight in bold the results for the two SNPs that showed association*.

Even though rs2708928 and rs3730364 are located in intronic regions, we decided to investigate possible functional implication by predicting the impact on protein function using six algorithms present in the Ensembl Variant Effect Predictor (VEP) (42), which included PolyPhen2 (43), Sort Intolerant from Tolerant (SIFT) (44), MutationTaster (45), PROVEAN (46), Combined Annotation Dependent Depletion (CADD) (47), and Functional Analysis through Hidden Markov Models (FATHMM) (48). However, we did not find evidence of a functional effect for either rs2708928 or rs3730364.

### *IL1B* RNA Quantification in Hippocampal Tissue

**Figure 1:**
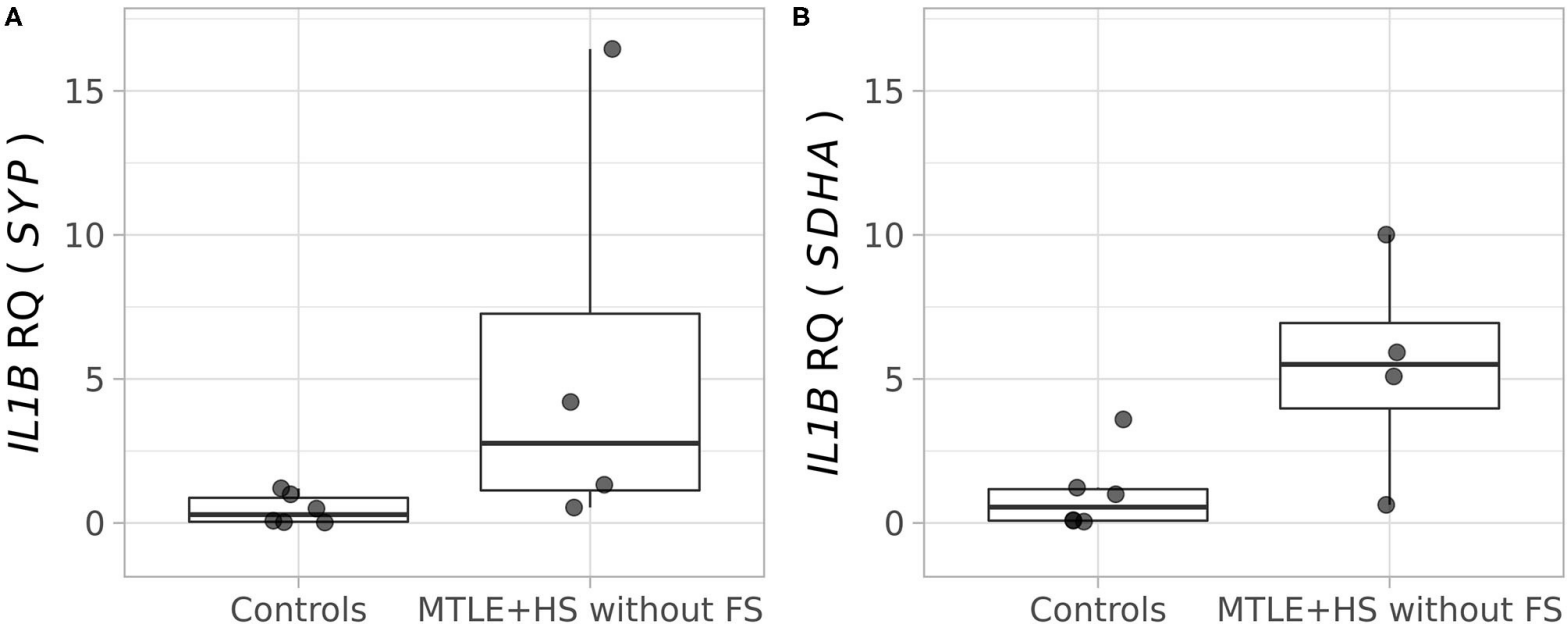
Relative quantification of the *IL1B* (interleukin 1 beta) transcript in the hippocampal tissue of four patients with pharmacoresistant mesial temporal lobe epilepsy, who were operated on to control refractory seizures, and autopsy controls (*n* = 6). **(A)** Analysis performed using the *SYP* (synaptophysin) gene as an endogenous control (Wilcoxon rank-sum test, adjusted *p* = 0.0762). **(B)** Analysis performed using the *SDHA* (succinate dehydrogenase, subunit A) gene as an endogenous control (Wilcoxon rank-sum test, adjusted *p* = 0.1334).

We did not find differences in the *IL1B* RNA transcript levels in the hippocampal tissue from patients with MTLE+HS, none of whom had an antecedent of FS, compared with control tissue using the two different endogenous control genes: *SYP* (Wilcoxon rank-sum test adjusted *p* = 0.0762; Figure 1A) and *SDHA* (Wilcoxon rank-sum test adjusted *p* = 0.1334; Figure 1B). For the transcript analysis, we used two different reference genes, *SYP* and *SDHA*, which have been validated specifically for this type of analysis in human hippocampal tissue (Figure 1) (33).

### IL-1β Protein Quantification in Plasma

We obtained information regarding the time of the last seizure before the blood collection in 52 patients and depressive symptoms in 87 patients. The proportion of patients who had seizures <24 h and more than 24 h before the blood collection was similar in patients with and without FS (Fisher's exact test *p* = 1.000; Supplementary Table 2). Also, the proportion of patients with depressive symptoms was similar in patients with and without FS (Fisher's exact test *p* = 1.000; Supplementary Table 3).

**Figure 2:**
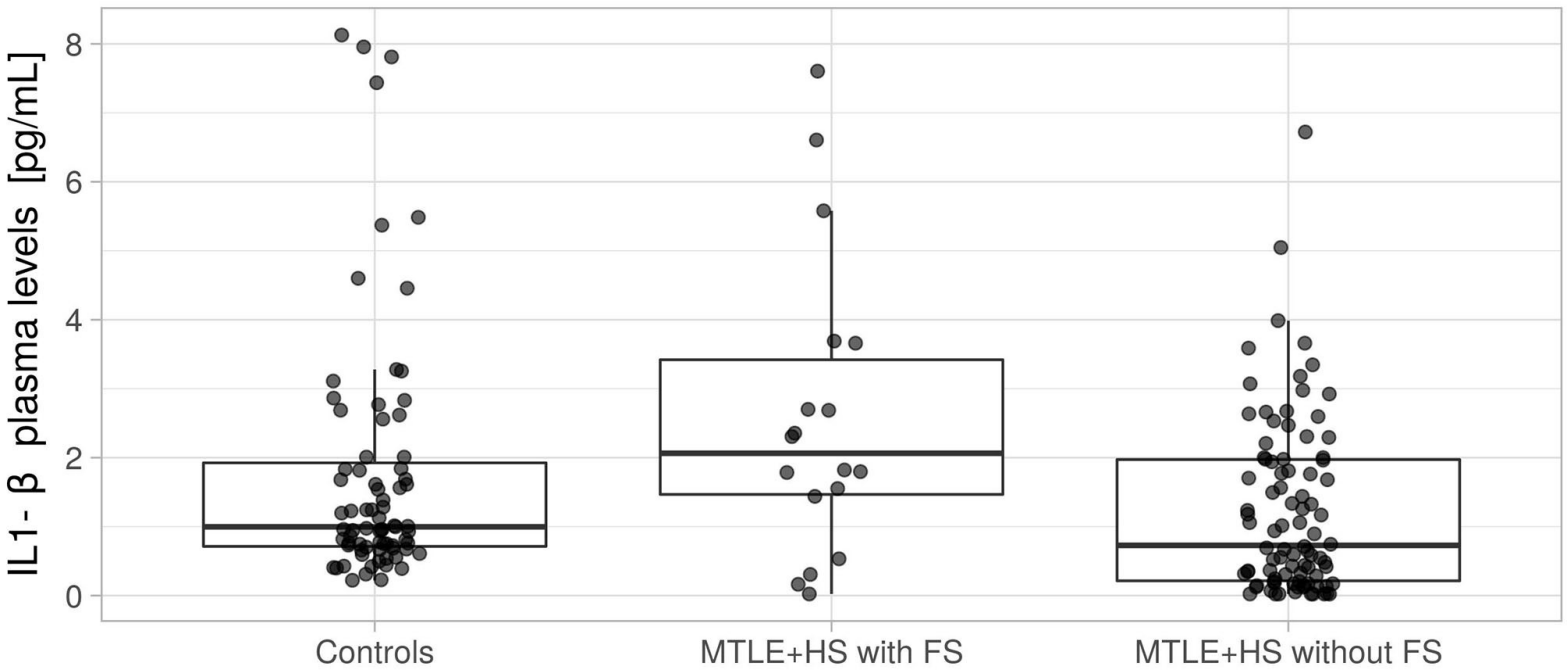
Quantification of interleukin 1 beta (IL-1β) protein in plasma from controls, patients with mesial temporal lobe epilepsy with an antecedent of febrile seizure (MTLE+HS with FS), and patients with mesial temporal lobe epilepsy with no antecedent of febrile seizure (MTLE+HS without FS). We used the Wilcoxon rank-sum test to compare patients with MTLE+HS with FS and controls (*p* = 0.1639) and compare patients with MTLE+HS without FS and controls (*p* = 0.0195).

As shown in Figure 2, the median IL-1β plasma concentration in controls was 0.997 pg/ml (25% quantile = 0.713 pg/ml, 75% quantile = 1.924 pg/ml). A similar concentration was observed in patients with MTLE+HS without FS (median = 0.728 pg/ml, 25% quantile = 0.214 pg/ml, 75% quantile = 1.973 pg/ml; Wilcoxon rank sum test *p* = 0.4539), including a small effect size (*r* = 0.129, 95% CI = 0.00–0.283). However, we observed a significant increase in the IL-1β plasma concentration in patients with MTLE+HS with an antecedent of FS (median = 2.063 pg/ml, 25% quantile = 1.466 pg/ml, 75% quantile = 3.420 pg/ml; Wilcoxon rank sum test *p* = 0.0097), including an increase in the effect size (*r* = 0.202, 95% CI = 0.052–0.347).

## Discussion

IL-1β is a powerful pro-convulsant and may have implications in epileptogenesis (8). In the last two decades, many studies have contributed to our knowledge about the role of the proinflammatory cytokine IL-1β in MTLE+HS and FS. However, there are still many controversies, and the subject is far from being settled (11–13, 15, 49, 50). Noteworthy, most previous studies investigating the role of IL-1β have not addressed the specific questions of whether IL-1β may be involved in the pathogenesis of MTLE=HS (10–16). Also, the work presented here can only indicate a link between IL-1β and MTLE+HS, and it was not explicitly designed to investigate causality.

We performed a candidate gene association study using SNPs encompassing the entire *IL1B*, including the still disputed associated functional variant−511C>T, present in the promoter region (20). This variant was first associated with MTLE+HS in a Japanese cohort (20) and Finnish children with FS (21). However, the association was not confirmed in other studies, for example, by Buono et al. (22) in a cohort with European ancestry, by Jin et al. (17) in Chinese individuals, and by Chou et al. (44) in Taiwanese children with FS. In addition, a meta-analysis revealed a modest association and effect size for the c.-511C>T polymorphism in patients with MTLE+HS (51). In the present study, we found no association between the c.-511C>T (rs16944) promoter variant in patients with MTLE+HS, with or without an antecedent of FS (Table 3). Of note, Kanemoto and coworkers reported that the rs16944^*^T allele might be a risk factor for prolonged febrile seizures (PFS) (52). Furthermore, several authors have hypothesized that c.-511C>T (rs16944) may increase *IL1B* transcription and the protein levels during PFS, elevating the risk of hippocampal injuries (14, 53, 54), which, in turn, could lead to the future development of MTLE+HS. Unlike FS, which are seizures that last <10–15 min in infants and young children and are believed not to be associated with an increased risk of epilepsy later in life, a history of PFS (lasting > 15 min) has been associated with subsequent MTLE+HS (55–57). However, determining the exact duration of an event that occurred decades earlier is either impossible or imprecise in retrospective studies, which explains, at least in part, the high variability in the proportion of PFS reported in the MTLE series (55–57). We could not unequivocally distinguish between FS and PFS in our cohort, which is a limitation of the present study. Thus, the absence of genetic association with the c.-511C>T (rs16944) variant found in the current study may reflect phenotypic differences in the cohorts.

Nevertheless, we found a genetic association signal between MTLE+HS and the SNPs rs2708928 and rs3730364 (Table 3). Interestingly, the SNP rs3730364^*^C, associated with an increased risk effect for MTLE+HS, appeared at a frequency of 0.673 in patients with MTLE+HS with FS and 0.414 in the control group. These frequency estimates are much higher than what was reported among 257 Brazilian exomes by the BIPMed project (rs3730364^*^C = 0.002; https://bipmed.org/) (58), as well as in the 1,000 Genomes (rs3730364^*^C = 0.008) and GnomAD (rs3730364^*^C = 0.007) databases (59, 60). We did not find the reported frequency for the SNP rs2708928 in public databases. SNPs rs3730364 and rs2708928 are located on the second and third introns of the *IL1B* gene, respectively, and according to prediction algorithms, there is no evidence that they have functional implications (61). Therefore, it is more plausible that both SNPs are in linkage disequilibrium with another functional variant, still unidentified, located within the *IL1B* gene.

In genetic association studies, addressing population stratification is a pivotal step to avoid spurious results due to genetic diversity between the compared groups (37, 38). Our AMOVA results showed a high variation component within groups, patients, and controls and a low variation component between the two groups (Table 2). Therefore, the two groups are most likely derived from the same population and do not present population stratification, allowing unbiased comparisons.

We also investigated the transcript levels of the *IL1B* gene in hippocampal tissue from four patients with pharmacoresistant MTLE+HS, who had no antecedent of FS, and we found no difference when comparing with tissue from autopsy controls (Figure 1). These results are somewhat different from previous studies investigating gene expression in resected hippocampal tissue from pharmacoresistant MTLE+HS and experimental models of temporal lobe epilepsy, which have shown an increase in *IL1B* mRNA levels (16, 62–67). However, all previous studies mentioned above have a critical limitation since they did not evaluate patients based on the antecedent of FS (16, 62–67). Thus, our results bring a novel and important finding regarding the relevance of considering the antecedent of FS when analyzing the pattern of transcript expression in the hippocampal tissue of patients with MTLE. Noteworthy, we also did not find differences in IL-1β levels in the plasma of patients with MTLE+HS without FS (Figure 2), which agrees with our transcript results. Thus, taking all the evidence presented here, we may suggest that the difference between our results and previous studies is because our *IL1B* mRNA quantification was carried out exclusively in patients with MTLE+HS without FS. However, we also recognize that we could not obtain hippocampal tissues from patients who presented antecedent of FS, and further studies comparing hippocampal tissue from patients with MTLE+HS with and without FS are essential to investigate this issue further.

It is also worth mentioning that our gene expression study in hippocampal tissue was carried out using two different reference genes, which have been validated specifically for this type of analysis in human hippocampal tissue (33). This technical aspect is also a key feature of the present study since it considerably increases the reliability of the tissue transcript analysis. Indeed, a previous study of protein expression in hippocampal tissue showed different results when using two different antibodies for the same protein, pointing to the lack of reliability of the western blot experiment in detecting the tissue expression of IL-1β and suggesting that RT-PCR is the method of choice to evaluate gene expression (68).

Finally, we quantified IL-1β protein in the plasma of patients with MTLE+HS and controls. We found similar IL-1β levels when comparing patients with no antecedent FS and controls, which agrees with our *IL1B* mRNA quantification findings in the hippocampal tissue. Also, we observed increased IL-1β plasma levels in patients with MTLE+HS with FS (Figure 2). The IL-1β plasma level has been reported to be increased in the postictal period in patients with MTLE and associated with depression in these patients (69). However, the proportion of patients with depressive symptoms and the intervals between the last seizure and blood collection were similar in patients with and without FS. Thus, our results might indicate a chronic inflammatory state in patients with MTLE+HS with an antecedent of FS, which may be less intense, or even not present in patients with MTLE+HS without antecedent FS (70). Furthermore, it has been demonstrated that IL-1β levels may be significantly different in the cerebrospinal fluid, blood, and central nervous system tissue of the same individual (18, 68), emphasizing the relevance of studying these different biological compartments. In addition, we recognize that upstream and downstream regulatory targets of IL-1β should also be investigated in further studies, including in the pediatric population (8, 9, 21, 23, 24).

In conclusion, we sought to assess the role of the IL-1β in a cohort of patients with an in-depth phenotypic characterization and using a multidimensional approach to access genetic association, transcript, and protein quantification of IL-1β. Not surprisingly, our results support a complex relationship for IL-1β in the context of MTLE+HS, as demonstrated by the positive genetic association with two SNPs in the *IL1B* gene in patients with MTLE+HS independently of the presence of FS. Also, there was an increase of plasmatic IL-1β levels only in MTLE+HS patients with antecedent FS, suggesting that they may have an immune system more prone to overexpress inflammation. Overall, our results support the hypothesis of a genetic association between MTLE+HS with the *IL1B* gene.

## Data Availability Statement

The datasets presented in this study can be found in online repositories. The name of the repository and accession numbers can be found below: The European Molecular Biology Laboratory's European Bioinformatics Institute (EMBL-EBI) European Nucleotide Archive(ENA), https://www.ebi.ac.uk/ena/browser/home, acession numbers PRJEB44301 and PRJEB39251.

## Ethics Statement

The studies involving human participants were reviewed and approved by the research ethics committee of the UNICAMP and USP-RP, and written informed consent was obtained from all participants.

## Author Contributions

ROS and RS created the study design, conceptualized the work, and performed data acquisition and analysis. PB, MS-A, MA, CY, FR, TV, AS, AT, and FC participated in data acquisition and analysis. CM-M and IL-C conceptualized the work and served as principal investigators. All authors reviewed and approved the final version of the manuscript.

## Conflict of Interest

The authors declare that the research was conducted in the absence of any commercial or financial relationships that could be construed as a potential conflict of interest. The Handling Editor KV declared a shared affiliation, though no other collaboration, with one or more authors TV and AS at the time of the review.

## Publisher's Note

All claims expressed in this article are solely those of the authors and do not necessarily represent those of their affiliated organizations, or those of the publisher, the editors and the reviewers. Any product that may be evaluated in this article, or claim that may be made by its manufacturer, is not guaranteed or endorsed by the publisher.
