# Supplementary material for: Multidimensional Approach Assessing the Role of Interleukin 1 Beta in Mesial Temporal Lobe Epilepsy
Source: Front Neurol. 2021 Aug 5;12:690847. doi: 10.3389/fneur.2021.690847 (PMC8375265; doi:10.3389/fneur.2021.690847)
Supplement: Supplementary file 1 [file Data_Sheet_1.PDF]

Supplementary Material

Multidimensional Approach Assessing the Role of Interleukin 1 Beta in Mesial Temporal Lobe Epilepsy

Renato O. Santos, Rodrigo Secolin, Patrícia G. Barbalho, Mariana S. Silva-Alves, Marina K. M. Alvim, Clarissa L. Yasuda, Fábio Rogério, Tonicarlo R.Velasco, Americo C. Sakamoto, Antonio L. Teixeira, Luciano S. Queiroz, Fernando Cendes, Claudia V. Maurer-Morelli, Iscia Lopes-Cendes*

Supplementary Table 1. Minor allele frequency (MAF) and Hardy-Weinberg disequilibrium analysis (HWD) for the SNPs used for the population stratification analysis (genomic control).

SNP	Position (base pairs)	MAF	HWD p-value
rs4854183	3365278	0.225	0.0395
rs312966	21137711	0.496	0.1445
rs4670075	34161126	0.116	0.7827
rs7568053	38419704	0.319	0.6357
rs11675509	45969431	0.193	0.0083
rs7609407	73273000	0.273	0.0657
rs6707475	74483364	0.409	0.0374
rs412079	80240368	0.436	0.0107
rs6713136	107264124	0.254	0.0454
rs6716554	107924531	0.310	0.0008
rs7425100	136210221	0.038	0.0963
rs2195114	144706197	0.355	0.1156
rs6727767	150159527	0.311	0.0531
rs2909450	162011281	0.169	0.1944
rs6432729	162616505	0.374	0.4211
rs840706	176827844	0.141	0.0043
rs13008910	183195941	0.117	0.4479
rs17202235	207355467	0.243	0.3010
rs10209471	211295062	0.282	1.0000
rs1829611	211596566	0.171	0.3131
rs284552	216448321	0.064	0.6337
rs2070735	227312185	0.208	0.5246
rs10179086	231879499	0.286	0.0891

MAF= minor allele frequency; HWD=Hardy-Weinberg disequilibrium

Supplementary Table 2. Distribution of patients with MTLE+HS who had seizures less than 24 hours and more than 24 before the blood collection used for IL-1β protein quantification in plasma.

	Less than 24h	More than 24h	Total	p-value
MTLE+HS with FS	1 (1.9%)	8 (15.4%)	9 (17.3%)	1.000
MTLE+HS without FS	7 (13.5%)	36 (69.2%)	43 (82.7%)	
**Total**	8 (15.4%)	44 (84.6%)	52 (100.0%)	

Supplementary Table 3. Distribution of patients with MTLE+HS with or without depressive symptoms (DS)* who contributed with samples for IL-1β protein quantification in plasma.

	With DS	Without DS	Total	p-value
MTLE+HS with FS	2 (2.3%)	12 (13.8%)	14 (16.1%)	1.000
MTLE+HS without FS	12 (13.8%)	61 (70.1%)	73 (83.9%)	
**Total**	14 (16.1%)	73 (83.9%)	87 (100.0%)	

* We used the recommended cutoffs for the Brazilian population of the Beck Depression Inventory (BDI-II) [1] to determine the presence or not of depressive symptoms (DS). The presence of DS was defined as scores above 19 (scores of moderate [20-28] and severe [29-63] depressive symptoms were combined). Patients with BDI-II scores below 19 were considered as not having significant depressive symptoms (scores of no depressive symptoms [0-13] and mild symptoms [14-19 were combined).

Reference:

1. Gomes-Oliveira MH, Gorenstein C, Lotufo Neto F, Andrade LH, Wang YP. Validation of the Brazilian Portuguese version of the Beck Depression Inventory-II in a community sample. Braz J Psychiatry. (2012) 34:389–94. doi: 10.1016/j.rbp.2012.03.005
